# Relationship between psycho-physiological indicators and task performance under various indoor space designs for telecommuting environment by introducing mixed-reality

**DOI:** 10.1038/s41598-024-52291-1

**Published:** 2024-01-23

**Authors:** Kyung-Tae Lee, Ju-Hyung Kim

**Affiliations:** Department of Architectural Engineering, Hanyang University, 222 Wangsimni-ro, Science and Technology Hall, Seoul, 04763 Republic of Korea

**Keywords:** Physiology, Psychology, Engineering

## Abstract

The increase in telecommuting during COVID-19 and advances in digital technology have necessitated the establishment of guidelines for maximizing productivity through indoor space design for telecommuters. Additionally, understanding the physiological response of individuals working in indoor spaces has attracted attention. This study applied mixed-reality environment to alter the design of the indoor space in real-time, while monitoring the task performance and representative psycho-physiological indicators (electroencephalogram and heart rate variability) of 30 individuals with telecommuting experience. To this end, four tasks, including spatial memory, attention, execution, and working memory, were conducted, and the psycho-physiological data from these tests were statistically analyzed. The results revealed that the design of the indoor space did not affect the spatial memory; however, the parasympathetic nerves were stimulated in visually non-preferred spaces, thus reducing mental stress and leading to high efficiency in short-term work. According to the Yerkes-Dodson law, the working memory of an individual is generally efficient and physically stable over time if they adjust to a preferred or decision-making space. Thus, the future design of telecommuting spaces must consider the type of work being done, and guidelines for spatial design should be developed by recognizing the psycho-physiological status of users, while increasing efficiency.

## Introduction

The outbreak of the coronavirus disease (COVID-19) has triggered significant changes in the behavior of people in terms of work environment, as they have to stay indoors for longer periods compared to the pre-pandemic era. Moreover, the significance of indoor spaces has been emphasized even before the pandemic as people typically spend 90% of their lifetime indoors for reasons, such as service, knowledge work, or production^1,2^. As the lockdown and social distancing make it difficult for people to go outside for work, significant number of companies encourage employees to work from home, while maintaining productivity and professionalism^3^. However, in some cases, it is challenging to maintain proper work performance at home. Traditionally, telecommuting was believed to guarantee high productivity under normal situations. However, the COVID-19 period exposed telecommuters to stress due to special situations, such as child-care support, and family work violations in individual work^4^. Therefore, it is crucial for individuals to have a designated space for work during work hours. Currently, enhancing the productivity of work-at-home individuals through indoor designs that prioritizes human comfort and needs has attracted research attention^5,6^. Moreover, as smart work becomes increasingly prevalent, the construction of spaces that can handle both labor and rest is essential. In addition, consumers are becoming increasingly interested in the philosophy and cultural appreciation of indoor spaces, so telecommuting environments should be designed to be more sensory-centered and accommodating to human needs.

To understand the effects of indoor environment on task performance, Kim et al.^1^ investigated the relationship between work productivity and thermal performance. They found that there is a positive relationship between alertness and working memory under warm conditions (25.7 °C), whereas there is a negative relationship between executive ability and mental workload under cool conditions (17 °C). In addition, Duyan and Ünver^7^ investigated the effects of the color of the wall on the attention of students, and found that purple and red walls contributed to higher work performance. Regarding the size of space, Marchand et al.^8^ found that there is a negative relationship between listening and reading tasks in spaces with low ceilings. These aforementioned studies have identified the effect of each detailed indoor space composition on task performance, but studies on work productivity in a comprehensively designed space with various factors are few. Therefore, it is essential to design an optimal indoor space that considers these factors to achieve the highest work efficiency.

Banaei et al.^9^ reported that certain aspects of the interior design, such as color, wall material, and ceiling height, can affect the physical and mental health of humans. Researchers have investigated how people adapt to indoor environments using physiological indicators, such as electroencephalogram (EEG) or electrocardiogram (ECG). For example, white or green walls make a space appear wider, and higher ceiling heights can increase the ’resilience of users. These effects have been validated by measuring brain waves and observing a relatively large number of alpha waves, which indicated a relaxed feeling^10^. Araujo et al.^11^ conducted an experiment to understand the relationship between light and physical health, and found that heart rate variability (HRV) relaxation occurred under red light, whereas active heart rate emerged under white light. Although a number of studies have been conducted to propose the optimal interior design composition, only few indoor spaces with a favorable psycho-physiological effect on humans have been developed. To better understand the relationship between space and task performance, research should be conducted in environments where the individual feels a realistic sense of space. In this context, virtual reality (VR) and mixed reality (MR) can be used to create immersive environments that emulate actual space under controlled experimental environment. Several studies have demonstrated that when implemented, there is no notable distinction in the VR- and MR-created environment from reality. Puyana-Romero et al.^12^ extended the validation of VR technology to the evaluation of the urban environment using a head-mounted display (HMD) and a laptop or desktop computer, and found that there was no significant difference in the real and VR environments in the subjective evaluation results. Moreover, Hong et al.^13^ reported that the utilization of mixed reality technology in outdoor urban settings had no significant effect on the evaluation of the soundscape compared to the physical environment. These results suggest that sufficiently valid results can be obtained when VR and MR technologies are used for subjective evaluation. Overall, these results suggest the applicability of MR technology to progressive, practical research for identifying interactions between indoor space design and task performance.

Physiological indicators reflect the status and responses of the human body system, such as the autonomic nervous system, which consists of sympathetic and parasympathetic divisions. Visual stimuli induce responses and affect the physical stimuli and behaviors of individuals through stress^14^. The Yerkes–Dodson law^15^ indicates that the enhancement of the task performance under stress is proportional to physiological arousal up until a certain point, beyond which an increase in stress results in a decrease in performance. Therefore, variation in interior design, such as color and layout, can affect heart rate, stress levels, workload, and task performance^16^. To provide the optimal design for telecommuters, it is essential to understand their physiological indicators and work performance based on the indoor space design. Thus, further efforts are required to comprehend the state of end-users and the work efficiency in their preferred space.

The aim of this study was to examine the impact of various indoor space designs on physiological responses and task performance, and to explore the relationship between work efficiency and physiological indicators. To achieve this, this study utilized psycho-physiological indicators and cognitive tests to analyze the physiological effects and task performance of participants under various spatial compositions. First, the preferred and non-preferred spatial compositions of individuals were determined through a preliminary survey. Subsequently, an actual experiment was conducted on 30 participants using an MR technology, in which the virtual environment was adjusted into the actual room dimensions, and task performance scores were examined using conducting cognitive tests. During the tests, the ’EEG and HRV of the subjects were measured to obtain the psycho-physiological indicators in each room. Lastly, the differences and impacts of the indoor space on the physiological state were explored by applying various statistical method, such as normality analysis, repeated measures one-way analysis of variance (RM ANOVA), Friedman's test, and linear regression analysis. Moreover, correlations between task performance and psycho-physiological responses were analyzed using Spearman rho's analysis.

## Materials and methods

### Experiment environment

This study was conducted in a room-sized indoor space with the wall color, material, and ceiling height as the independent parameters of the experiment, and the study was conducted using MR technology. As MR was created by enclosing the virtual environment within the actual room dimensions, the virtual design was covered by the actual environment because humans must see and feel that area. This is because it will assist the experiment participants to intuitively feel the change in the design of the space composition. The indoor space used in this study was a room at Hanyang University with a size of 5500 mm (width) × 7000 mm (length) × 3600 mm (height). A table, chair, and laptop were used as the sample in the MR setting, and the experiment consisted of four situations: (1) a white environment as the basic space, (2) preliminary survey results of the preferred design space of the participants, (3) non-preferred design space of the individuals, and (4) the decision-making space. To prevent the ordering effect, multiple MR environments were listed in random order for each subject during the experiment in each space.

#### Pre-survey for interior design

To identify the preferred and non-preferred spaces of individuals to develop the MR environment, the color, wall material, floor material, and height of the ceiling, which are the parts of the interior space used in this study, were inquired in advance. Under the assumption that the people who conducted the survey were working from home in a single room, a space believed to have the highest work efficiency was constructed. A total of 5 points were reviewed, and 205 individuals in their 1920s–1930s, who had worked from home for more than a year due to COVID-19, were investigated. Through this, the color of the wall and floor, the material of the wall, the height of the ceiling and the preference for furniture arrangement were selected.

**Figure 1:**
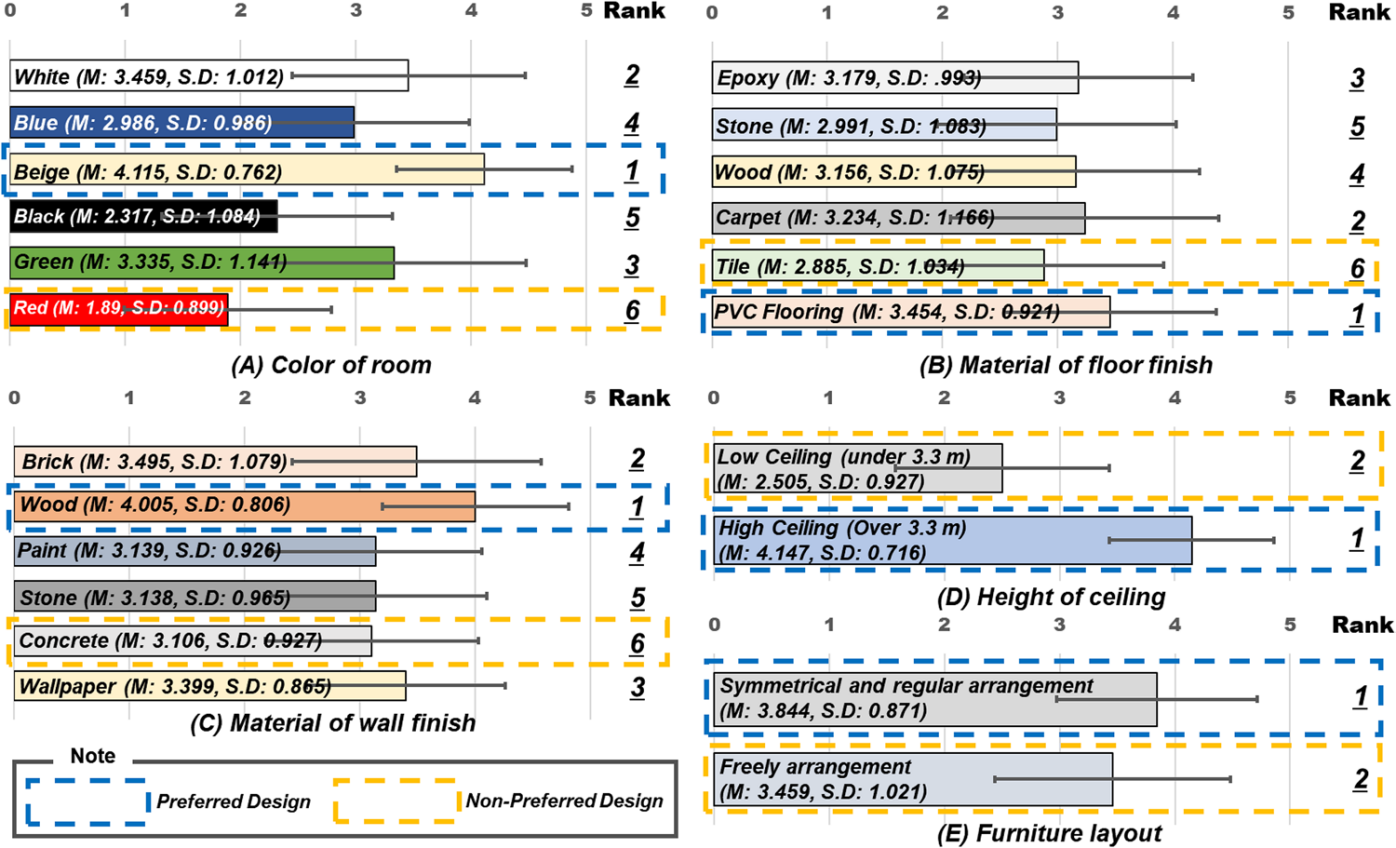
Preliminary survey results of the experimental space composition.

The result of survey is shown as the Fig. 1. People had the most positive perceptions for beige, Poly Vinyl Chloride (PVC) flooring, woody, high ceilings, and symmetrical environments. This indicated that work will go well in a comfortable warm atmosphere and well-organized space. In contrast, the appearance of the red concrete walls, and low ceilings in the form of tile finishes were considered undesirable. These conditions are believed to result in a low work efficiency, as these conditions visually stimulates and gives a stuffy feeling. Therefore, these two designs were utilized for the experimental environments to construct a virtual environment.

#### Experimental setting

To evaluate the physiological condition and the task performance in the experimental space, a MR environment was realized in the following way. First, the condition of room was easily and rapidly changed using the MR software programs SketchUp and Trimble Connect. The initial 3D model was constructed using SketchUp, after which the modeling file was loaded to Trimble Connect through the cloud system, and real-time rendering was performed to make the virtual environment seem more physically real. The dimensions of the 3D model are the same as that of the actual room. The temperature and humidity of all experimental rooms were adjusted to 23 ± 0.4 °C and 41 ± 2%, respectively, based on the ASHRAE guidelines to provide proper conditions for human occupancy^17,18^. To show the MR environment to experimenters, a representative hardware device, Hololens 2, was used. Hololens are made of glass on the front and hardware on the rear, allowing users to simultaneously encrypt real-world information and project holographic data on the display-to-display MR information on the glass^19^.

When the experiment began after the subjects wore the Hololens 2, they were asked to select the best individual space for work based on the items asked in the preliminary survey. Thereafter, four environments established with MR technology were shown for the experiments. During the experiment, an environment similar to the space in which work is performed was established by placing actual tables and chairs in the actual room. The virtual environment implemented a space similar to the real room. The researchers could obtain accurate physiological responses and task performance of the experimenters as there was no difference in the experiment environment of the actual space and the virtual images shown as holograms^18,20^. All the experiment process was performed in accordance with the relevant guidelines and regulations.

### Subjects

All the process and protocol were approved by the Institutional Review Board and Ethics Committee of the Hanyang University (HYUIRB-2022202-005). The experiments were conducted using 30 healthy peoples with experienced home-work for at least 1 year during COVID-19 to obtain the right subjects for the purpose of this study. The number of subjects in the experiment was calculated through G-power 3.1, to reveal the adequate sample size based on previous similar studies^9^. The subjects consisted of 15 males (50%) and 15 females (50%) with an average age of 27.13 years, and the standard deviation of the age was 2.55 included in the early adulthood (age 22–34 years). All the subjects had no any medical history of chronic diseases, such as eye disease, visual impairment, and mental illness. Before the experiment, the subjects were asked about the quality of their sleep the previous night. Moreover, caffeine or alcohol intake and smoking was prohibited in the last 24 h. All the experimenters participated voluntarily, and obtained informed consent before enrollment to the study, while their anonymity has been preserved throughout the duration of the study. The subjects who did not satisfy the required conditions were excluded.

### Experimental protocol

**Figure 2:**
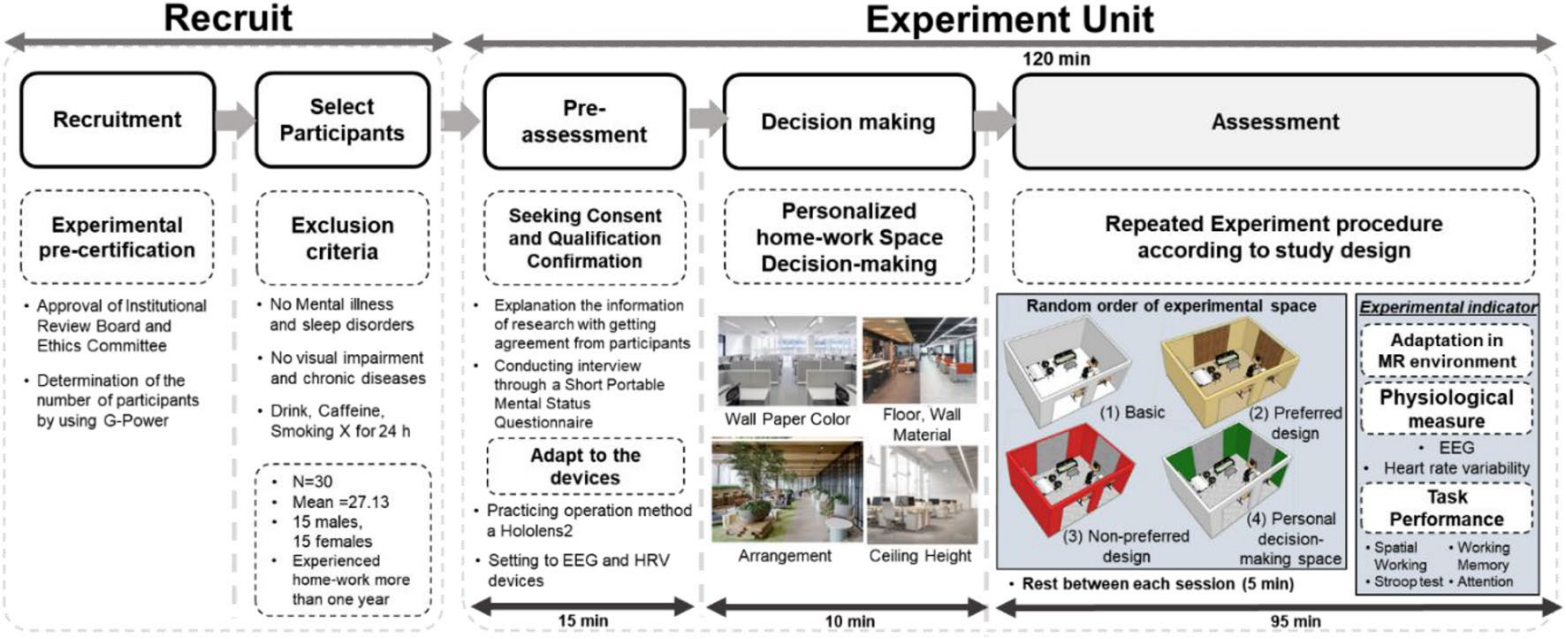
Experimental protocol.

Figure 2 shows the experimental protocol of this study: (1) pre-assessment, (2) Decision-making, and (3) measurement.

**Figure 3:**
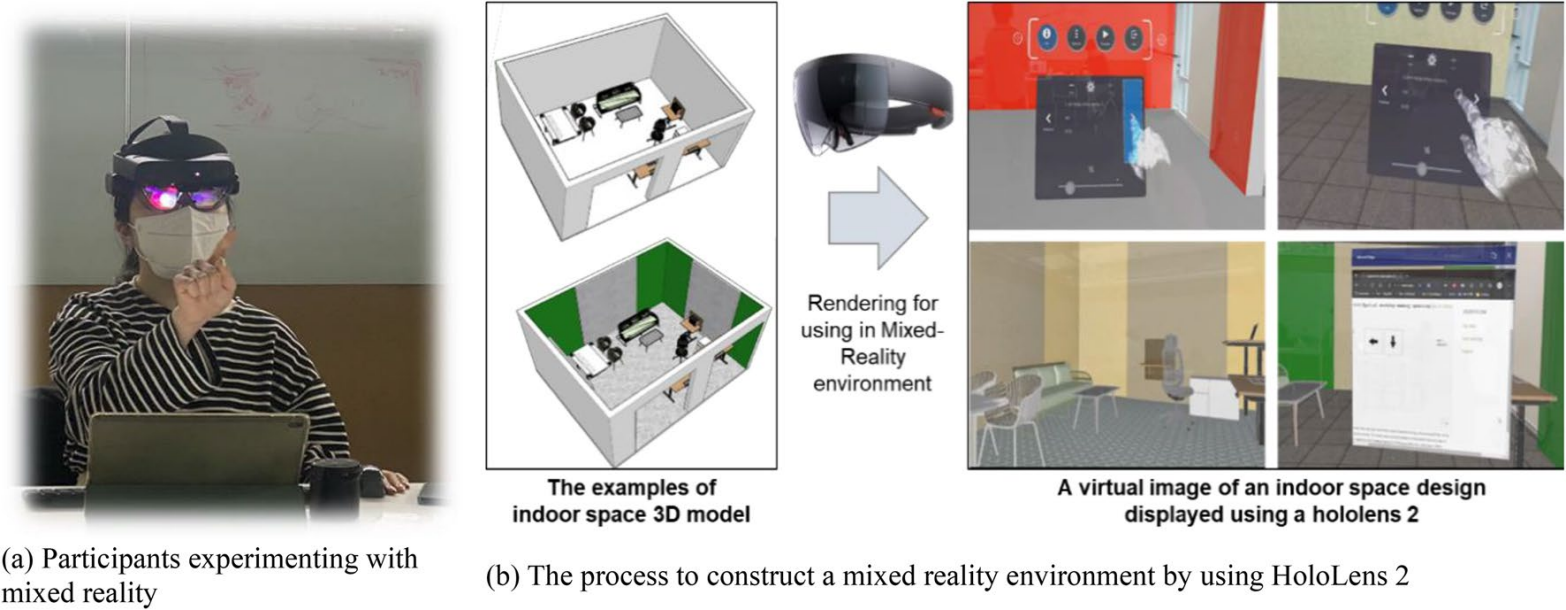
Description of the hardware utilized in the experiment and the surrounding environment.
- **Step 3. Measurement (100 min):** In this experiment, the conditions of the four rooms are shown randomly to minimize the systematic experimental bias. The experiment was conducted in each virtual space for 20 min, with a 5-min break after each session. When conducting each experiment, despite constructing the environment based on the real room, the virtual environment was suddenly covered, so that it was first adapted not to feel dizzy. Thereafter, the four cognitive tests were performed using the Hololens 2 to identify task performance, while intuitively showing the virtual interior design. The EEG and HRV data were measured simultaneously from each task.

- **Step 1. Pre-assessment (10 min):** The process of this study was explained verbally using organized documents. Short Portable Mental Status Questionnaire (SPMSQ) was distributed to the subjects, and they were asked to select their perception status^21^. When the participants were deemed fit to participate in the experiment, they were trained on how to operate Hololens 2 to adapt the MR environment. Cognitive test was performed using a computer to prevent learning effect^18^. Moreover, the subjects were set to adapt to the devices prior to the study to obtain the EEG and HRV data for this research.
- **Step 2. Decision-Making (10 min):** The subjects were asked to wear the Hololens 2 on their head, after which they were asked to construct an indoor space that seemed to be the most efficient for work. Figure 3a is the image of the participant included in the study and obtained informed consent to publish the image in publication. Participants selected the space they wanted by changing options in the order of wall color, floor and wall material, and ceiling height, (Fig. 3b).

### Assessments

#### Assessment of the physiological responses of the subjects

It is well known that the physiological indicators of a person change with a change in their environment^11,16^. In this experiment, EEG and HRV were used as physiological indicators, and the physiological response of the subjects was measured using an EEG device by recording the brain waves from their cerebral cortex. An EEG is a non-invasive technique for recording the brain electricity signal and feedback of the activities of brain nerve cells, and they are obtained from electrodes attached to the scalp. This device enables the real-time observation and high temporal resolution of brain activity when changes in voltage from neurons are detected by the electrode. Moreover, EEG features in the frequency domain are relatively notable and helps in the identification of the relationship between emotions and architectural space at a low cost and high time resolution^1^. In this study, brainwaves were recorded using Emotiv EPOC + composed of 14 channels of active electrodes based on the international 10–20 system. This device can obtain brain activates from four lobe: (1) frontal lobe: AF3, AF4, F3, F4, F7, F8, FC5 and FC6; (2) temporal lobe: T7 and T8; (3) occipital lobe: O1 and O2; and (4) parietal lobe: P7 and P8 (Lievesley, Wozencroft and Ewins, 2011). Obtaining the values from these lobes helps in the prediction of emotions and behaviors of each subject, and shows the working state of the human brain^22,23^.

Compared to EEG, HRV is a non-invasive method for quantifying the cardiac autonomic function. The heart is connected to the autonomous nervous system (ANS) through the vagal and sympathetic and parasympathetic nerve fibers. Changes in nerve activity and the release of hormones affect cardiac activity and provide biofeedback^24^. When the heart beats, the sensors receive electrical impulse and blood flow, and it is measured in time-domain results in milliseconds and frequency domain methods^25^. HRV influences activities, such as exercising or resting, as it is significantly influenced by emotions and stress, it can be used to assess the emotional state and level of satisfaction in each place^26^. In this study, the Elite HRV CorSense gadget was employed to quantify the HRV. Elite HRV CorSense helps in obtaining HR in real-time with beat-to-beat intervals (R-R) time series as an output, which has ground truth compared to other devices, such as Polar H10 and other smart watches, owing to the high correlation coefficients^27,28^. The Elite HRV CorSense monitor reflects the adaptive ability of the ANS to respond to stressors acting on the pilot at any given moment. A photomicrosensor within the device detects signals in the fingertip area. The blood in this region reflects and scatters the emitted optical beam, causing a change in the optical energy detected by the sensor. This alteration corresponds to the rhythmic pulsation of blood in the capillaries^29^. Participants wore the CorSense on their second finger before proceeding with the experiment. To avoid noise data associated with movements and artifacts, the first five minutes of the recording were disregarded before obtaining the HRV data. For each detected R wave, R–R intervals (the time between two successive R waves) and the instantaneous heart rate were obtained. The data were then collected using the Elite HRV application and utilized for downstream analyses.

#### Assessment of the Task analysis of the subjects

**Figure 4:**
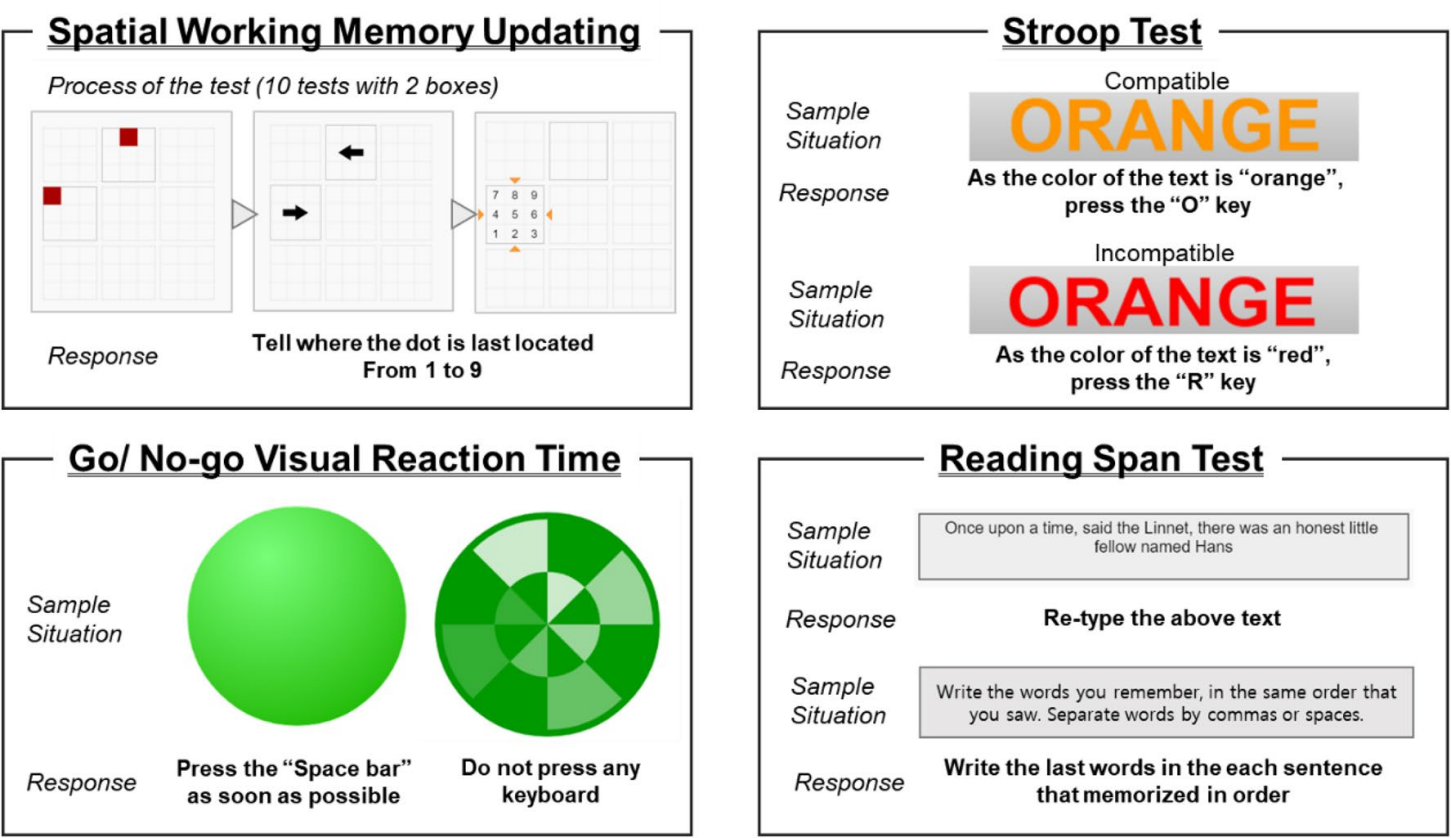
Cognitive tests for identifying the task performance under different interior designs.

All the subjects were required to participate in the cognitive tests for 15 min using the device under each of the interior design space situation. The cognitive test is an analytical method that allows for the assessment of the participant’s performance and comprehension of their tasks while observing their neuro-behavioral reactions, including decision-making, identification, perception, and working memory^1^. Additionally, cognitive tests are employed to evaluate the concentration of an individual and serve as indicators of future job performance. Given the challenges in directly assessing work efficiency, these tests serve as substitutes to rapidly gauge how people's brains operate^30^. Despite the numerous aspects of brain function assessable through cognitive tests, this study specifically used four cognitive tests, illustrated in Fig. 4, to determine task performance: (1) the Spatial Working Memory Updating test, (2) Stroop test, (3) Go/No-go visual reaction time, and (4) Reading Span Task. These activities effectively serve as tests to illustrate states such as workload and stress that individuals experience during work. Notably, cognitive functions such as working memory, attention, and executive function, which are evaluated through activities such as the Mini-Mental State Exam-Korea and Literacy Independent Cognitive Assessment in South Korea, allow for the assessment of not only cognitive abilities and daily life activities but also of work efficiency^31^. The experiments utilized similar tests to those employed in prior studies, ensuring comparable competencies for the purpose of this study. Previous research has assessed each individual’s capacity to judge and evaluate the indoor environment using visual tasks and tests, including reading comprehension, the Stroop test, and working memory^1,18,30,32^. Additionally, while experiencing each room presented in a mixed-reality environment intuitively, it enabled focused on the work in a visually visible setting, creating the impression of a genuine work situation, and enhancing immersion and realism. In this study, the participants practiced the following tests in advance to reduce the influence of learning during the experiments.

- **Spatial working memory updating test:** This test measures the spatial working memory of the participant based on the spatial memory test developed by Salthouse et al.^33^. For this test, the participants were presented with a 3 × 3 matrix composed of blank squares displayed in the screen and the dot started from random positions. Next, the participants were asked to remember the positions of the dots, and follow their positions as they move around the cell as indicated by the arrows. After moving the dots several times using the arrow, participants were requested to select the final positions of the dots. In addition, they have to speak as fast as possible and were required to solve 10 questions. This determines whether the subject receives sensitive information in space and solves the issue^34^.
- **Stroop test:** This test measures the executive ability of the participants. It aims to evaluate the accuracy and pace in writing the first letter of the color shown. The dissonance between the color presented and the mismatched name interferes with naming the color. For example, when the screen displays the color yellow with the word "green," the subject is expected to press Y rather than G. It causes elevated activation in the cingulate cortex included in detecting the incongruity.
- **Go/No-go visual reaction time:** This test measures the attention ability of the participants. For this test, the subjects were instructed to press the spacebar as fast and as accurately as possible when the plain dot was displayed. However, if the checker patterned dot is shown, they were requested to ignore the sign. In other words, response to the alternate stimulus needs to be inhibited.
- **Reading Span task:** This test measures the working memory of the participants. As individuals have different working memory and reading skills, this test analyzes the working memory capacity, as well as reading comprehension ability, and the performance was also correlated with verbal speaking performance^35^. In this test, the participants were required to type the three complete sentences displayed on the monitor screen as quickly as possible and to memorize the final word of each sentence. Afterwards, if the subject successfully types all three words, the question is finished. The objective was to solve three similar problems.

In these tests, response time (milliseconds) and accuracy (percentage) were identified as the main indicators for assessing the learning performance. This study calculated the working performance by integrating and evaluating these indicators along with 0.5 geometric weighting based on Eq. (1).

1 $${\text{Learning Performance}} = \left[ {\left( {{\text{Accuracy}}} \right)^{{0.5}} *\frac{1}{{\left( {Response\;time} \right)^{{0.5}} }}} \right]^{2} *100$$

### Pre-processing of physiological data

#### Pre-processing of EEG signal

After importing the EEG data to EEGLAB using MATLAB, we loaded the electrode position information. First, the basic poser of the wave and its source location were identified. The EEG features in the time domain are not notable, and needs to extract features using algorithms^36^. However, the machine used to measure EEG is very susceptible to almost all stimuli, such as psychological and sensory input, breathing, emotions, and limb movement. In addition, unwanted artefacts, such as eye flicker and muscle movement, could hinder the accurate measurement of the brainwaves. Therefore, it is important to effectively remove artefacts and noise in the early stage of the analysis through signal preprocessing. First, raw EEG signals of specific frequency range (2–39 Hz) were passed through a finite-impulse response (FIR) filter. The FIR filter has various transfer functions, and previous studies have applied Blackman, Hamming, Hann and Rectangular window to analyze EEG data. EEG windowing type has a different normalized transition width value^1,37,38^. This study utilized Blackman window with minimal side lobe and outstanding frequency resolution. Thereafter, de-noise methods were applied to remove the remaining artefacts using independent component analysis (ICA), and it enables the separation of the artefacts and the real-brain activity. Lastly, to analyze EEG band power, fast Fourier transform (FFT) was performed to classify the signals into theta wave (4–8 Hz), alpha wave (8–13 Hz), beta wave (13–30 Hz), and gamma wave (30–39 Hz). Theta waves are primarily produced during sleep; alpha waves are generally emitted when the brain is at rest; beta waves appear while the brain is at alert, working, or being active; and gamma waves are emitted when there is intense excitement or concentration. Nonetheless, as absolute intensity varies from person to person, the analysis must be conducted using the value transformed to relative band power.

In this research, the physiological responses of the subjects were assessed using two electroencephalographic indices. Although there are various formulae, such as Alpha-1 and 2, Task Load Index, this study utilized equations that can determine the mental workload and mental stress when operating in space^39,40^. Equation (2) expresses the calculation of the mental workload, whereas Eq. (3) expresses the calculation of the mental stress value.

2 $${\text{Mental workload }} = \frac{{(Relative\;Beta)_{{Frontal}} }}{{(Relative\;Theta)_{{Frontal}} + (Relative\;Alpha)_{{Frontal}} }}$$

3 $${\text{Mental Stress}} = \frac{{(Relative\;Theta)_{{Frontal}} }}{{(Relative\;Alpha)_{{Parietal}} }}$$

#### Pre-processing of the HRV

Given that the indoor environment, device configuration, and individual factors can influence the variability of HRV signals, we initially undertook data preprocessing. Frequency cut-off values were defined to automatically eliminate outliers and ectopic beats from the entire dataset. In this study, these values were set at 0.05–0.15 for LF and 0.15–0.4 for HF using the FFT method (FFT; 1024 points) in Matlab. Data points with detection values outside these ranges were replaced with NaN values. Following the removal of artifacts and poor signal data, HRV analysis was performed using the Kubios HRV software (Version 4.0.2; https://www.kubios.com/download/). This analysis adhered to specific criteria: (1) Gaussian R–R intervals and heart rate distribution graphs; (2) absence of large R–R interval outliers; (3) and equidistant R–R intervals. The R–R intervals series were detrended using “smoothness priors” with the alpha value set at 500, and correction analysis was conducted by applying a cubic spline interpolation through the software’s automatic method^41^.

The time-domain methods were directly introduced to the series of consecutive RR interval values^42^. Based on the RR interval and mean of RR, we computed the root mean square of the successive differences (RMSSD [ms]), the standard deviation of all normal R–R intervals (SDNN [ms]), and the number of pairs of adjacent normal R-R intervals differing by more than 50 ms divided by the total number of all RR interval (pNN50 [%]). These parameters are physiologically explained by fluctuations in the sympathetic and parasympathetic neural systems associated with mental state and mood changes^43^. Although there were significant changes in the sympathetic nervous system while concentrating, the parasympathetic nervous system is active in resting and stable states^42^. The SDNN, which is similar to total power in frequency domain, reveals the periodic fluctuations in the heart rate (both long and short-term variation of heart rate), and presents the activity of the sympathetic nervous system^44^. RMSSD enables the identification of the short-term variation in the heart rate, and measures the parasympathetic activity to identify physiological stress^45^. The pNN50 reflects the activity of the parasympathetic nerve during a brief period of time, and the larger the variance in the adjacent interval, the greater the value, and the more physiologically healthy the individual is^46^. Similar to RMSSD, pNN50 is also closely correlated with the degree of the parasympathetic nerve activity, and studies have demonstrated that increased RMSSD and pNN 50 are associated with feelings of calm and relaxation^43^.

Frequency-domain features are derived from the power spectrum density^45^. We obtained the power in the high frequency (HF: 0.15–0.4 Hz [ms^2^]) and the low frequency (LF: 0.04–0.15 Hz) via estimation using auto-regressive (AR) modelling, which is divided into distinct spectral components by applying spectral factorization^42^. LF reveals how sympathetic and parasympathetic nerves influence the HRV signals, whereas HF reveals the variance of HRV for parasympathetic nerves^47^. The power ratio, which is LF is divided by HF (LF/HF), reflects the degree of the balance between the sympathetic and parasympathetic nerves, hence indicating the control of the cardiovascular autonomic nerve. Consequently, the increased stress results in decreased HF, increased LF, and a greater LF/HF ratio^39,44^.

### Statistical approach

The experiment employed a repeated-measures design that allows one subject to perform cognitive tests sequentially in four different interior design of an indoor space with the same conditions. To confirm that the changes in each space are statistically significant, owe-way repeated-measures analysis of variance (RM ANOVA) was performed. The procedures of the one-way RM ANOVA are as follows: (1) normality (2) homoscedasticity of the dependent variable, and (3) sphericity. However, if normality was not satisfied, one of the non-parametric statistic method, Friedman’s test, was performed^18^. First, even though the 30 subjects participated in this study, as required by the *Central Limit Theorem* to assume normality, tests of normality, such as the Kolmogorov–Smirnov test (K–S test) and the Shapiro–Wilk test, were conducted. In this research, to compare the results according to the interior design, physiological indicators, such as mental stress and pNN50 and task performance satisfied normality (p > 0.05 in K–S test), whereas, mental workload, RMSSD, SDNN and LF/HF did not. Next, to examine the homoscedasticity of the dependent variable, Box’s covariance matrix was applied, and the p-values are all greater than or equal to 0.05 for factors that satisfied normality, so the null hypothesis is satisfied. Lastly, the assumption of sphericity was performed through the Mauchly’s test. If it was violated (p-value < 0.05), Greenhouse–Geisser was introduced to correct the degrees of freedom^48^. The significance level of the one-way RM ANOVA and Friedman’s test was expressed as a p-value, and a value of less than 0.05 (p < 0.05) indicates that there is a statistically significant difference between the groups. Additionally, post-hoc analysis was conducted to determine which of the groups exhibited a significant difference. For this research, Tukey’s honestly significant difference (HSD) was adopted for RM ANOVA and Bonferroni correction was adopted for the Friedman’s test. This method is commonly employed owing to the advantage of comparing small groups, which was intuitive^18,26^. Accordingly, as there are factors that did not satisfy the normality, Spearman rho correlations were employed to examine the correlation between physiological responses of EEG and HRV data in the indoor space. In addition, the physiological parameters that were significantly affected by each task performance were evaluated using the multiple linear regression model. The measurement of multi-collinearity was based on the variance of inflation factor (VIF) and standardized coefficient beta was obtained to compare the strength of each independent variable to the dependent variable^49^.

## Results

### Differences in the physiological data according to the spatial design

#### Spatial comparison of the EEG-based statistical information

For each spatial design, the mental stress and mental workload were compared based on the obtained value. If the mental stress satisfies normality (K–S > 0.200), it was investigated using RM ANOVA, whereas the mental workload was assessed using Friedman's test with a K–S probability of significance of 0.030. Prior to analyzing the mental stress, sphericity test was conducted for each test. The Mauchly's test revealed that sphericity was presumed for most of the indicators except the working memory span, the. Therefore, the working memory span was analyzed using Greenhouse–Geisser Epsilon.

**Figure 5:**
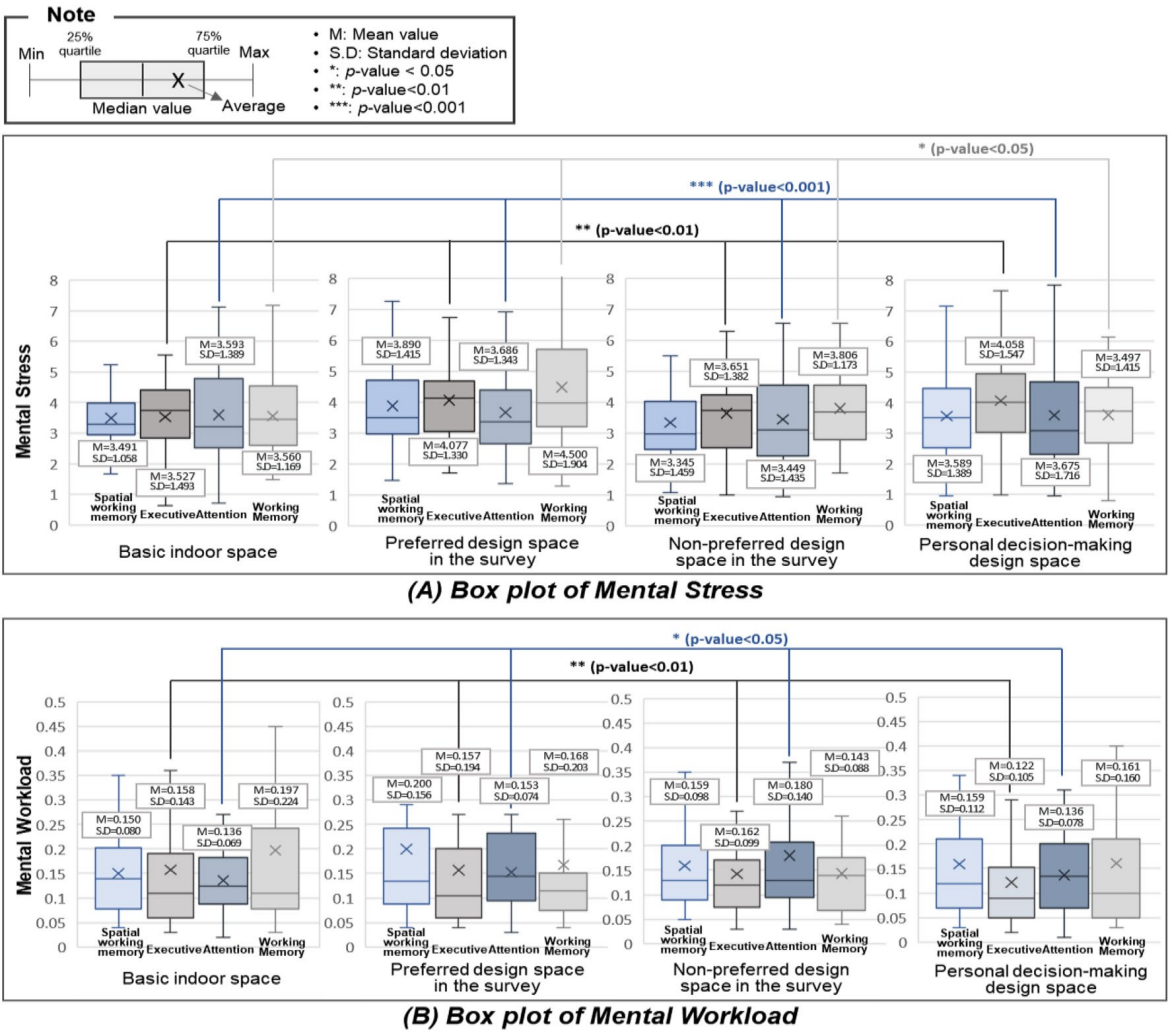
Boxplot of the EEG with alteration in the indoor space design.

Table 1 shows the EEG results according to the indoor space and Fig. 5 shows the boxplot showing the mean values and standard deviation of the mental stress and mental workload under the four experimental conditions. When the mental stress was analyzed using one-way RM ANOVA, the results revealed that there were significant differences in the executive ability, attention, and working memory with a change in the indoor space design. In addition, the examining tests with substantial variations revealed that the highest mental stress was observed in the most preferred space, whereas mental stress was lowest in the non-preferred space. Tukey's post-hoc test revealed that there were statistically significant differences between the executive competence, attention, and working memory. In terms of the executive ability and working memory, the mental stress was highest in the non-preferred space. For the attention variable, the highest mental stress was observed in both non-preferred and preferred environments.

**Table 1:** EEG results of the test with a change in the indoor space design.

EEG results	Test	Tests of within-subject effects (one-way RM ANOVA)					Post hoc analysis
		Type III sum of squares	df	Mean square	F-statistics	Significant	Tukey HSD
Mental stress	Spatial working memory	5.011	3	1.670	1.369	0.258	–
	Executive ability	6.780	3	2.260	1.320	0.003**	1 = 3 < 4 < 2
	Attention ability	1.981	3	1.776	1.412	0.001***	1 = 3 < 2 = 4
	Working memory	12.344	2.222	5.577	3.481	0.033*	1 = 4 < 3 < 2

	Test	Experiment space	Mean rank	Friedman’s test		Post-hoc analysis	
				Chi-square (χ^2)^	Significant	Assessment place	p
Mental workload	Spatial Working memory	Basic (1)	2.60	1.716	0.465	–	–
		Prefer (2)	2.70				
		Non-prefer (3)	2.33				
		Personal (4)	2.37				
	Executive ability	Basic (1)	2.60	5.234	0.006*	1–2 1–3 1–4 2–3 2–4 3–4	0**** 0**** 0**** 0**** 0**** 0****
		Prefer (2)	2.50				
		Non-prefer (3)	2.25				
		Personal (4)	2.65				
	Attention ability	Basic (1)	2.50	4.280	0.035*	1–2 1–3 1–4 2–3 2–4 3–4	0**** 0**** 0**** 0**** 0**** 0****
		Prefer (2)	2.50				
		Non-prefer (3)	2.23				
		Personal (4)	2.77				
	Working memory	Basic (1)	2.37	3.560	0.216	–	–
		Prefer (2)	2.50				
		Non-prefer (3)	2.07				
		Personal (4)	2.47				

*p < 0.05, **p < 0.01, ***p < 0.001, ****post-hoc analysis adjusted p < 0.0083.

A non-parametric Friedman’s test of differences among repeated measures of mental workload of each test in each space was conducted. The results revealed that there was no significant difference in the spatial working memory and working memory with a change in the indoor design, whereas there was a significant difference in the executive ability and attention ability. The average rank indicates that the mental workload in the non-preferred space was substantial. In contrast, there were significant differences between the executive ability and attention with a change in the indoor space, and the workload in the individual-determined space was the lowest. Post-hoc tests were performed by applying Dunn-Bonferroni corrections, which is the Mann–Whitney test conducted for each group, and the significance level was divided by the number of Mann–Whitney tests when there is a significant difference. As four groups were investigated in this study, the Mann–Whitney test significance level was 0.05/6 = 0.0083. Consequently, there were differences in the executive and attention abilities in all groups with a change in the space design. Thus, the pressure from work increased in the least preferred space with a change in the space design owing to the high mental burden, whereas less strain from work was felt in the preferable space owing to the low mental workload.

#### HRV data statistics results

**Figure 6:**
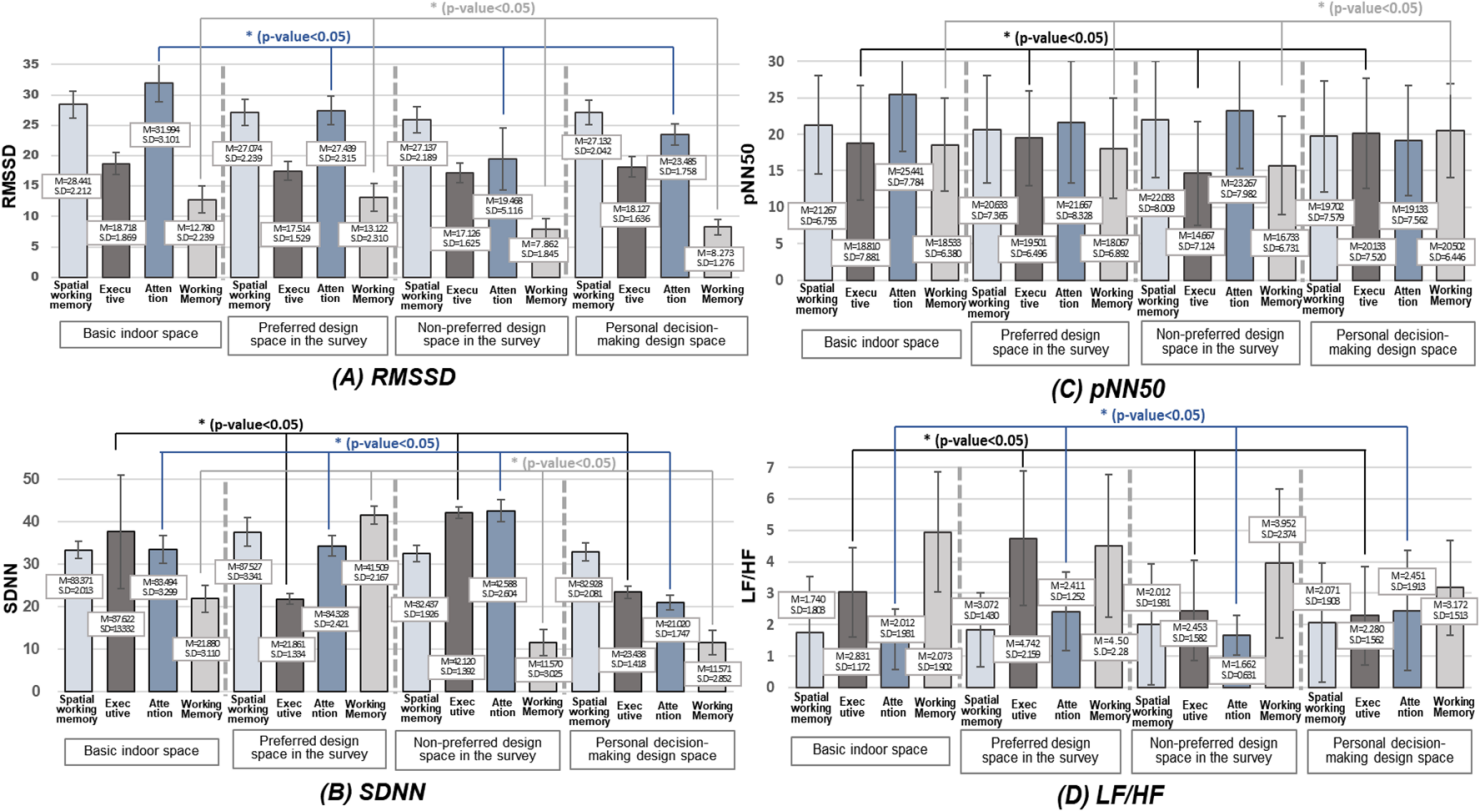
Boxplot of the HRV with a change in the indoor space design.

In this study, the RMSSD, SDNN, pNN50 in the time-domain methods, and LF/HF in the frequency-domain methods, were utilized as indicators for identifying the HRV results by indoor space. Based on the K–S test, RMSSD (p = 0.000), SDNN (p = 0.001), and LF/HF (p = 0.032) were compared using Friedman’s test, and pNN50 (p = 0.152) was obtained using one-way RM ANOVA. The results are shown in Fig. 6. Figure 6 was described based on the raw data and analysis results after performing the non-parametric analysis.

The non-parametric analysis revealed that the average RMSSD reduced in the order of basic interior space, preferred space, decision making space, and non-preferred design space, indicating that the RMSSD in the non-preferred space was low, whereas the RMSSD in the basic space was high. Owing to the activation of parasympathetic activity in the basic and preferred space, this indicates a rather relaxed and calm state. After performing the Friedman test, and analyzing its results, we observed that there was a significant between the attention ability and working memory. In addition, the Bonferroni post-hoc test revealed that there was a statistically significant difference in the attention capacity across all spaces. However, there was no significant difference between the working memory in a basic space and the preferred space, and in the non-preferred space and the decision-making space, confirming that all other spaces are distinct.

When the ranking average for SDNN was calculated using non-parametric statistics, the ranking in the non-preferred space was observed to be the highest. In contrast, the individual decision-making space ranked low in terms of the spatial working memory and executive ability, and the basic indoor space ranked lowest in terms of attention and working memory. The evaluation of the SDNN of each space revealed that there was a statistically significant difference between the variables in each space except the spatial working memory. The SDNN value of the executive and attention ability in the non-preferred space was high, whereas the SDNN value in the personal decision-making space was low. The Bonferroni post-hoc test revealed that there was no significant difference in the execution ability in basic space and non-preferred space, but there was a difference in preferred and decision-making space. In the case of attention ability, there is no notable difference in the attention ability in basic and preferred space. Additionally, there was no notable difference in the working memory in non-preferred space and the decision-making space.

The RM ANOVA analysis of pNN50 revealed that there were significant differences between the executive ability and working memory in each space. Analysis of studies showing significant differences revealed that the PNN50 values for both executive ability and working memory was the lowest in non-preferred space, whereas they exhibited the highest values in the personal decision-making space, indicating the activity of the parasympathetic nerve and a physiologically stable environment. The post-hoc analysis revealed that there was no significant difference between the basic space and the preferred environment, but that the place where personal decisions were made was advantageous.

Lastly, the LF/HF research revealed that the average ranking of LF/HF was highest in the non-preferred space. Thus, the LF/HF ranking was generally at highest in the non-preferred space as a whole. According to the Friedman test, there was a substantial difference in the spatial working memory and attention ability with a change in the space design. The Bonferoni test revealed that the spatial working memory varied across all spaces. In addition, it may be seem that there are distinctions between all non-preferred attention spaces, but there was no difference between basic and decision-making and preference and decision-making spaces.

### Statistical analysis of the task performance for each space design

As the interior design was altered, RM-ANOVA was conducted for each test that could satisfy normality in all task performance (Special working memory: 0.100, Executive ability: 0.200, Attention ability: 0.111, and Working memory: 0.178). Spatial working memory and attention ability assumed sphericity, and other tasks were analyzed using Greenhouse–Geisser Epsilon. There was a notable difference in these variables with a change in the spatial design (Table 2). The Tukey HSD analysis revealed that except the working memory, the efficiency of other factors was relatively higher in the non-preferred space than in other space designs. Additionally, except the spatial working memory, the efficiency of other factors was the second highest in the decision-making space.

**Table 2:** RM ANOVA results of task analysis.

Task performance (different interior design)	Interior design	Mean	Standard deviation	Tests of within-subject effects					Post hoc
				Type III sum of squares	Df	Mean square	F-statistics	Sig	Tukey HSD
Spatial working memory updating	Basic (1)	1.717	1.324	39.975	3	13.325	9.547	0.000**	1 = 4 < 2 < 3
	Prefer (2)	2.959	1.685						
	Non-prefer (3)	3.186	1.729						
	Personal (4)	2.949	1.506						
Executive ability	Basic (1)	9.349	4.063	549.384	2.572	213.564	27.966	0.000**	1 = 2 = 4 < 3
	Prefer (2)	12.203	3.960						
	Non-prefer (3)	14.790	4.088						
	Personal (4)	12.275	4.044						
Attention	Basic (1)	11.377	2.049	4.335	3	1.445	4.602	0.000**	1 = 2 < 4 < 3
	Prefer (2)	11.253	2.048						
	Non-prefer (3)	14.762	1.707						
	Personal (4)	13.530	1.765						
Working memory	Basic (1)	1.046	0.753	2.319	2.600	0.892	2.815	0.042*	3 = 1 = 2 < 4
	Prefer (2)	1.055	0.656						
	Non-prefer (3)	0.916	0.660						
	Personal (4)	1.301	0.492						

*p-value < 0.05.

**p-value < 0.001.

The Tukey HSD analysis revealed that work efficiency in the non-preferred space was high when the working memory was excluded. There was no significant difference in the spatial working memory, executive ability, and working memory in the basic space and the individual decision-making space. Moreover, there was no difference in the attention in the basic space and the preferred space, but was higher than those in the decision-making space and non-preferred space. The non-preferred space was observed to be advantageous for spatial memory recognition and simple short-term tasks, whereas the preferred space was advantageous for working memory, a long-term task.

### Results of Spearman rho’s correlation analysis

To determine the correlational coefficients among the eight factors (refer to Table 2) whose statistical significance had been verified, Spearman’s rho was performed after converting the total value into the t-score. The T-score enables the comparison of the values on the same line and standardization based on the average of 50 and standard deviation of 10. Spearman's rho's correlation was performed to analyze the correlation. The correlation coefficient was divided into the following five levels. When the absolute value of the coefficient is (1) 0–0.2: weak correlation; (2) 0.3–0.4: fair correlation; (3) 0.5–0.6: moderate correlation; (4) 0.7–0.8: strong correlation; and (5) more than 0.8: perfect correlation^50^.

**Figure 7:**
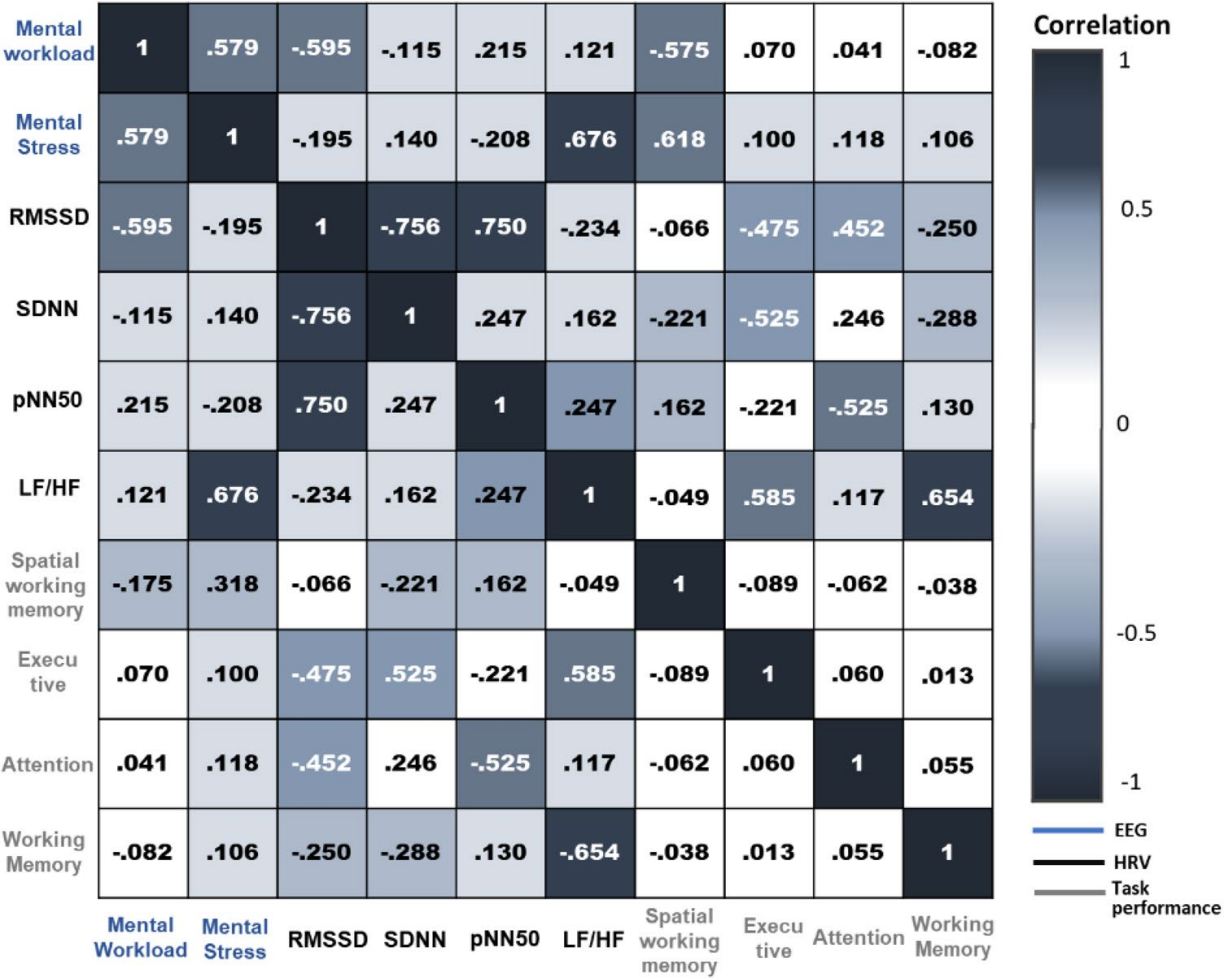
Results of correlation analysis between physiological indicators and task performance.

The correlation analysis used in this study was based on the experimental space with a relatively high task performance, which is the non-preferred space. Figure 7 shows the results of the correlation analysis, with the white letters representing static significance for both sides. When the physiological indicators were first analyzed, a correlation between mental workload and mental stress was observed. In addition, RMSSD was observed to be strongly correlated with SDNN and pNN50. Moreover, as mental stress is strongly linked to LF/HF, the relationship between RMSSD and pNN50, which influence each other via parasympathetic nerves, was observed to be strong. As the stress caused by visual stimulation generated in the non-preferred space increased, the LF increased and the HF decreased, resulting in an increase in the LF/HF value. However, a negative correlation was observed between mental workload and RMSSD.

The task performance result revealed that there was only a slight correlation between tasks. In addition, there was no significant correlation between the spatial working memory and physiological indicators. For the executive ability, there was a negative correlation between its RMSSD and pNN50, a positive correlation between SDNN and LF/HF, and a negative correlation between the RMSSD and pNN50 of attention. In conclusion, there was a strong negative correlation between the working memory and LF/HF. Therefore, in the non-preferred space, tension and fatigue are reduced during short-term tasks, such as execution and attention, but sympathetic activity increases during long-term activities, resulting in poor performance.

### Multivariate regression model

To understand the impact of the physiological indicators on task performance, a multivariable regression analysis was conducted. As shown in Table 3, based on the RM-ANOVA and Friedman's test results, only factors with significant differences were included in the regression analysis. The regression analysis was conducted using the results of the non-preferred space and the decision space, which exhibit significant differences among variables. In the case of executive and attention ability, the results of the non-preferred space with the highest workability were analyzed, whereas for the working memory, the results of the personal decision space were used.

**Table 3:** Psycho-physiological indicator exhibits significant space-dependent differences for regression analysis.

Independent variable	Dependent variable					
Task performance	EEG		HRV			
	Mental stress	Mental workload	RMSSD	SDNN	pNN50	LF/HF
Spatial working memory	–	–	–	–	–	–
Executive ability	0	0	0	0		0
Attention ability	0	0	–	0	0	–
Working memory	0	–	0	0	0	0

“0” indicate variable with significant difference.

First, three regression models were investigated using Durbin–Watson (DW) test. The DW test was performed to examine autocorrelation in the residuals from a regression model, and all the obtained values were between 0 and 4. A value of 2.0 indicates that there is no autocorrelation in the data^9^. All the DW statistic exhibited near-2 values (Executive: 1.879, Attention: 1.972, Working memory: 1.609), indicating that there was almost no auto-correlation in residuals. Subsequently, VIF was obtained for multicollinearity analysis. A VIF value of below 5 does not indicate a high correlation and does not require any measure to remove collinearity^51^. All the VIF values were lower than 10, indicating that there is a low correlation between the independent variable.

**Figure 8:**
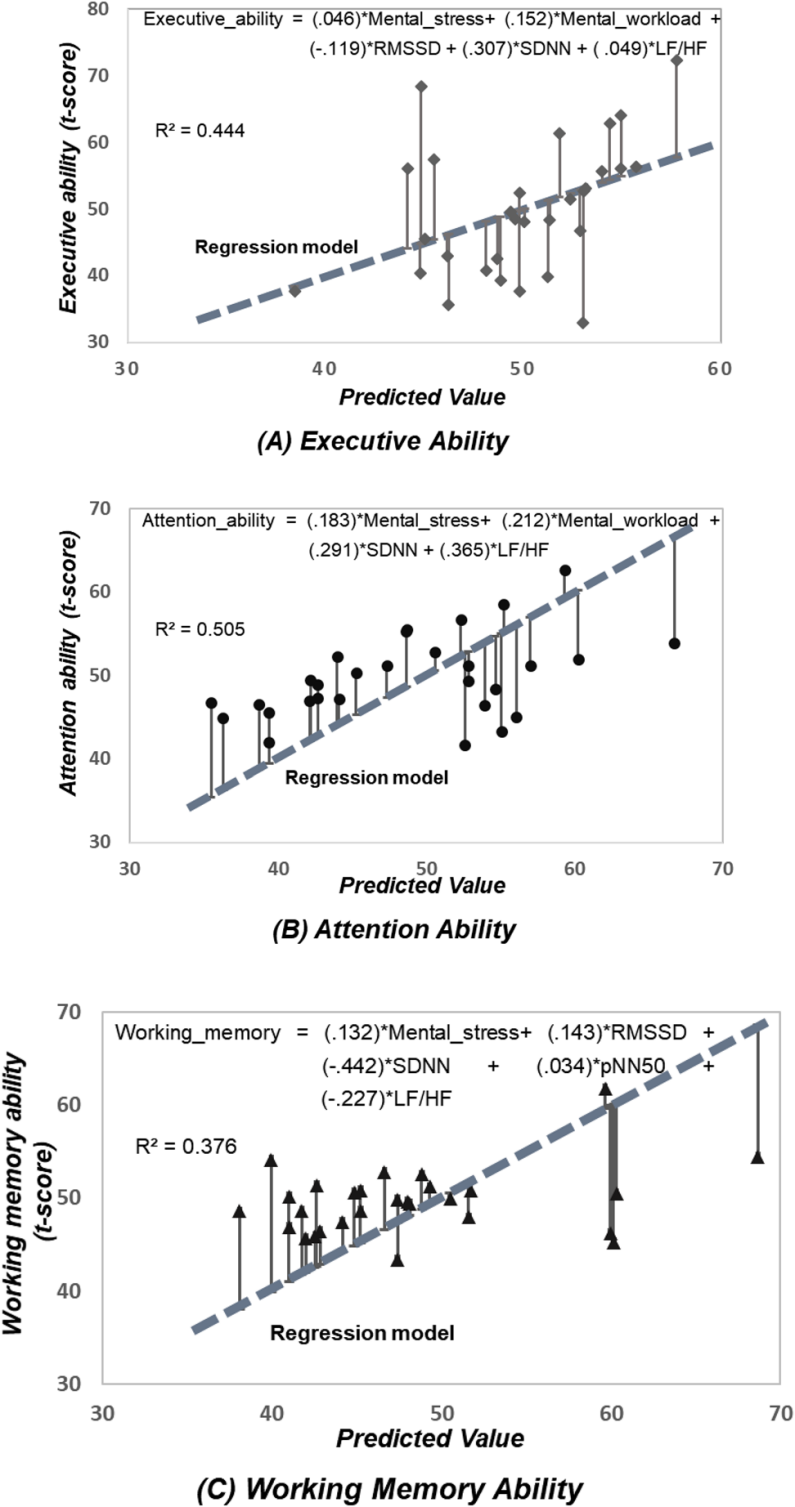
Results of linear regression model and equation for each task.

Figure 8 shows the results of the equation showing the degree of prediction by examining the impact of the independent variable on the dependent variable for each linear regression model. The SDNN was observed to exert the greatest positive influence on the factor, whereas RMSSD exhibited the most negative influence on the execution ability. In addition, the mental stress and LF/HF were confirmed to exhibit slight influence. For the attention ability, LF/HF, SDNN, mental workload, and mental stress exhibited the highest quantitative impact in that order. Lastly, SDNN was demonstrated to exhibit the greatest negative effect on the working memory abilities, whereas LF/HF exhibited the greatest positive influence. In contrast, pNN50 exerted slight effect.

## Discussion

**Figure 9:**
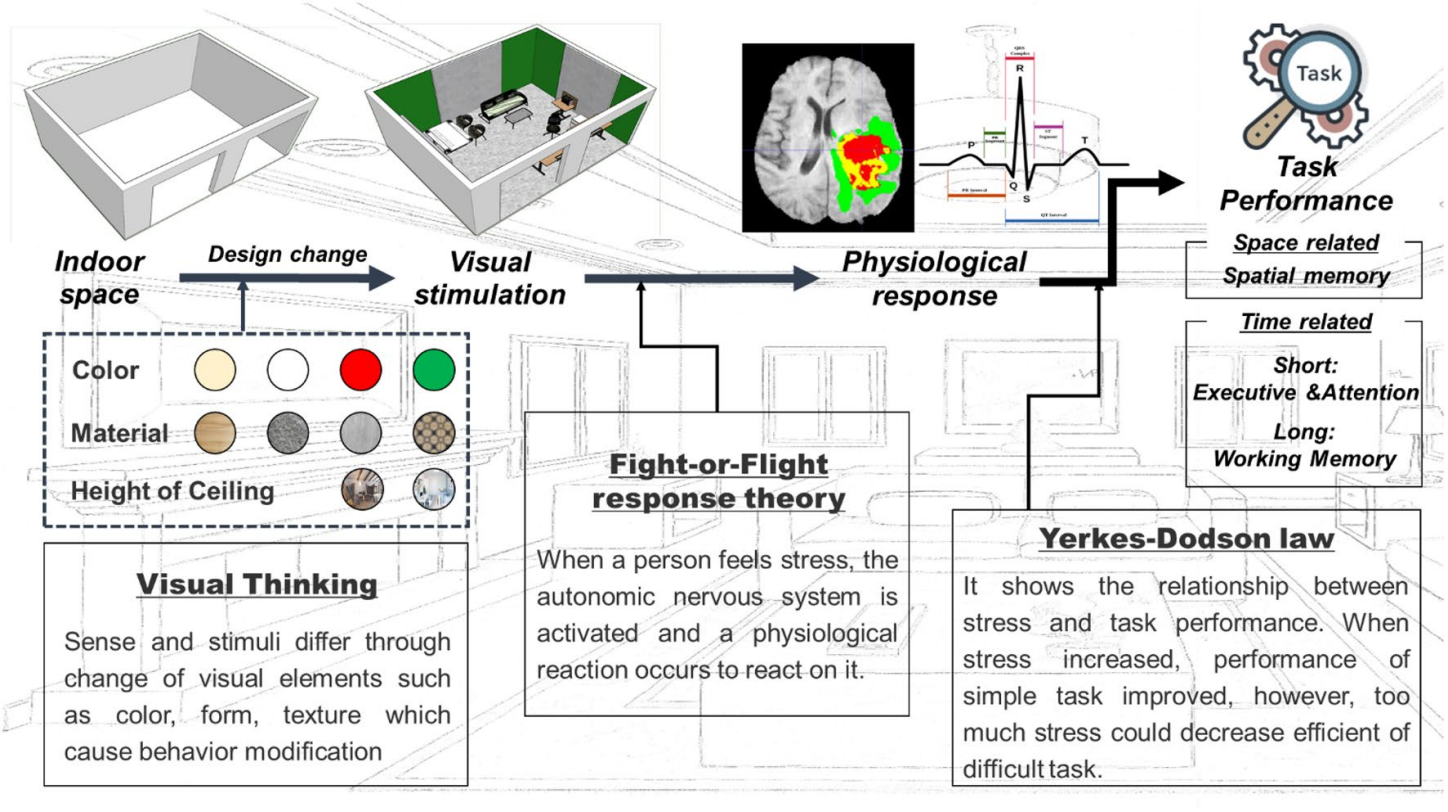
Physiological effects of change in the indoor space design and task performance.

As the COVID-19 outbreak has increased the home-work culture, this study investigated the correlation between task performance and psycho-physiological indicator with a change in the interior design using various statistical analysis. The task performance was determined using slightly cognitive test and the neuro-behavior and the EEG and HRV-based physiological indicator were identified^1^. These findings were utilized to determine the relationship between the visual stimulus of space and psychophysiological effect of the interior design on the task performance (Fig. 9). This study aims to propose the physiological indicator that should be considered to create an indoor space with high work efficiency for the development of interior space design.

### Physiological indicators based on interior design for improved task performance

This study presents four work space environments which cause physiological strain. From the psycho-physiology perspective, the change in the spatial design perceived in the mixed-reality environment experimented through HoloLens2 causes visual thinking. This implies that a perception of change of a person is influenced by visual elements, such as color, behavior, texture, and space pattern, and that these elements induce a change in the stimulus felt^52^. Moreover, individual preferences and non-preferences were found to influence the stimulus, as the space identified in the preliminary survey and the area of individual decision-making ultimately modify the stimulus based on visual thinking^14^. As illustrated in Fig. 1 and consistent with earlier studies, beige and white settings were favored for conveying a sense of friendliness and tranquility, while stimulating hues were deemed negative and ineffective^53^.

The results of this study indicate that psycho-physiological situations and behaviors can be determined using Visual stimulation^5,54^. As shown in Table 1, there were changes in the EEG with a change in the interior space design, and it can be demonstrated that short-term task performance (i.e., executive capacity and attention) influences both mental stress and workload. Similarly, RMSSD (Fig. 6a) and pNN50 (Fig. 6c) exhibited averagely low values among HRV markers. Consequently, the parasympathetic nerve activation was observed to be low. This phenomenon can be explained using the fight-or-flight response theory also known as acute stress response^55^. This theory states that the autonomous nervous system is activated when humans feel stress as it focuses on responding to the stressful situation. In other words, when exposed to indoor environments, such as red walls and low ceilings, the stress level increased significantly, and the attention and executive function were observed to improve rapidly^7^. The mental stress and LF/HF were also quite low, and it was discovered that even when the parasympathetic nerve was substantially activated, the sympathetic nerve was similarly activated. Consequently, even if space-generated stress exists, adaptation in space occurs throughout time.

What appears as a consequence of adaptation is task performance. As shown in Table 2, among the cognitive tests conducted in this study, there was a significant change in the physiological markers with a change in the indoor space in terms of executive and attention abilities requiring rapid performance. Nevertheless, there was no significant change in the autonomic nervous system associated with long-term working memory. This was also observed in the findings of the regression analysis. In the non-preferred space, SDNN exerted significant influence on the executive ability and attention, with the highest impact on task performance. In the case of working memory, however, SDNN and LF/HF were observed to exert detrimental effects and reduce sympathetic nerve activity. This could be explained by Yerkes-Dodson law, which states that performance improves with an increase in the stress levels until it reaches a peak, beyond which it remains constant, whereas the efficiency of complicated tasks diminishes progressively, resulting in a significant decline in performance. In the short term, discomfort in the non-preferred environment causes high focus, but in the long run, the balance between sympathetic and parasympathetic nerves occurs, and the task itself is more responsive to stress than the space.

In addition, based on the findings of the study, there is no significant difference in the physiological changes associated with the decision-making, preferred space, or intellectually preferred space of an individual. As presented in the “Results” section, as the difference in physiological indicators according to space has low significance and minimal correlation with the spatial working memory, there was no significant change with a change in space. However, despite the benefit of low-preference places for jobs that can be performed in a short amount of time, it is vital to create a preferred location as work productivity improves with time and physiological signs are expressed positively.

### Limitation and recommendations

This study aimed to explain and describe physiological change with a change in the interior designs in a small room with a mixed environment comprising of real and virtual images. By understanding the task performance in each environment, the changes with a change in the interior space design can be understood. However, further studies are needed to address the limitations of this study, which are as follows. First, the findings of human physiological indicators are objective, but additional research related to subject perception and emotion from these indoor spaces is essential by applying techniques, such as Pleasure-Arousal-Dominance^9^ and Positive Affect and Negative Affect Schedule^18^. This will enable a clearer understanding and analysis of person's emotions based on the interior space and the relationship between visual cognition and individual perception. In addition, as individual experiences and experiences affect human visual perception, it is vital to comprehend the experiences of the participants to comprehend spatial decision-making^4,14^. Secondly, experiments were carried out in a virtual environment with multiple devices activated, and experimenters and supervisors were connected one-on-one to conduct the experiment. There were challenges in maintaining complete concentration on the situation. Therefore, the findings should be validated in a more realistic environment and under representative disciplinary conditions. Thirdly, the condition of the subjects on the day before the experiment was not documented through subjective questionnaires. Factors such as the hours of sleep the night before, the time since the last meal, and the hours since the last alcohol consumption can significantly impact the performance of the subjects. Therefore, in future research, we plan to measure the self-reported sleepiness and blood alcohol concentration of the subject. Fourth, age may also be an important factor. In this study, the majority of participants were in their twenties and thirties, however their favorite place varied based on their age and task performance^5,19^. In the future, we intend to perform a complex analysis with similar early adherence and middle age propositions (35–64). Moreover, as there may be potential factors (cultural, educational) that could have influenced the results of the study, a further study controlling such factors, such as using people from a common background as much as possible, will be conducted.

For the real-life application of the findings of this study, the following steps must be suggested. First, the indoor space should bey realized and validated by performing various activities in it. Owing to the constraints of directly feeling and perceiving space in virtual settings, it is essential to collect objective psychophysiological signs, subjective opinions, and emotional data regularly. In addition, if results are produced by analyzing work performance based on actual tasks as opposed to simplified cognitive tests, a design that promotes work productivity during telecommuting can be constructed.

## Conclusion

This study examined the effects of different interior designs on the task performance and physiological indicators. In addition, the relationship between task performance and the physiological response under different interior design was investigated using various statistical analysis techniques. To this end, MR-based environment was employed to provide systematical and immersive considerations for experiments. Interior design of indoor space was more sensitively experienced through the Hololens2 than in the monitor to similarly experience a realistic situation through virtual images in a real environment. Consequently, the spatial working memory, executive feasibility, attention, and working memory could be distinguished through the use of physiological indicators and EEG- and HRV-based cognitive tests.

The key findings of this study are summarized as follows: (1) The results of the four tests revealed that the task performance varied with a change in the space, and that the efficiency of the individuals was high in all non-preferred environments, except the working memory, (2) The short-term task analysis revealed that there was a significant difference between the mental stress and mental workload, and mental stress is low in non-preferred area, whereas the mental effort is high, (3) Except for the inconsequential tests, the average cognitively normal pNN50 in the most of the non-preferred HRV region was low, the mean value of RMSSD was modest, and the mean value of SDNN and LF/HF was large, (4) Although the spatial working memory exerted less influence, it appears that stress occurred because the executive ability and attention exhibited a positive correlation with LF/HF and pNN50, and the working memory exhibited a positive correlation with LF/HF, (5) In addition, the regression test results revealed that SDNN exerted a statistically significant positive influence, and the quantitative influence of SDNN and LF/HF was demonstrated for the attention variable, indicating that the sympathetic nerve activity was high and mental effort was also substantial. In addition, SDNN and LF/HF exerted a significant negative effect on the working memory ability, and (6) Spatial alterations generate visual thinking, particularly in spaces that individuals perceive to be unpleasant. Under this condition, stress is induced in the short term, the autonomic nervous system is active, with increased focus on short-term task, and task performance is high. In contrast, over time, not only adaptation to the preferred area, but also task performance in the personal decision-making space were observed to be high.

This study confirmed that changes in the design of an interior space affect the task performance and physiological changes. Although prior studies have explored the link between interior design and the emotional state and mental status of individuals or have explored human states in response to alterations in thermal conditions or spatial configuration^5,56^, this study provides more comprehensive results by evaluating both human affective psycho-physiological states and work efficiency based on spatial design. Consequently, stress occurs owing to changes in the physiological indicators (EEG and HRV) with a change in the indoor space design. This influence is significant in the non-preferred space for short-term activities, and in preferred spaces for long-term activities. Furthermore, upon interpreting the research results, our study provides key insights into the effects of workplace characteristics on people by analyzing human states through both physiological and psychological lenses. This integrated approach provides key insights into the influence of architectural design on human well-being^1,5,19^. It is expected that the findings of the study can be applied to other studies on the creation of indoor space and what type of tasks can be performed efficiently with an increase in the number of telecommuting operations.

## Data Availability

The datasets generated and/or analysed during the current study are not publicly available due to possessing confidential information of the participants and the experiment was conducted under the assumption that their data would not be shared, but are available from the corresponding author on reasonable request.
